# No evidence for restriction of Beta-HPV8 gene expression by epidermodysplasia verruciformis susceptibility genes CIB1, TMC6, or TMC8 in keratinocytes

**DOI:** 10.1016/j.tvr.2025.200328

**Published:** 2025-10-22

**Authors:** Tina M. Rehm, Christina Parpoulas, Elke Straub, Thomas Iftner, Frank Stubenrauch

**Affiliations:** Institute for Medical Virology and Epidemiology of Viral Diseases, University of Tuebingen, Tuebingen, Germany

**Keywords:** Epidermodysplasia verruciformis, Beta-human papillomavirus, CIB1, TMC6, TMC8, Restriction factor

## Abstract

*Epidermodysplasia verruciformis* (EV) is an autosomal recessive disorder characterized by an extraordinary susceptibility to infections with human papillomaviruses (HPV), mainly from the genus beta. EV patients carry biallelic loss-of-function mutations in *TMC6* encoding Transmembrane channel-like protein 6 or EV protein 1 (EVER1), *TMC8* encoding Transmembrane channel-like protein 8 or EV protein 2 (EVER2), or *CIB1* encoding Calcium and integrin-binding protein 1. TMC6, TMC8 and CIB1 form a protein complex in the endoplasmic reticulum which has been hypothesized to act as a restriction factor for beta-HPV in keratinocytes. SiRNA-mediated knock-down of CIB1, TMC6, or TMC8 greatly reduces transcript and protein levels, but does not activate beta-HPV8 gene expression in normal keratinocytes. *TMC6* and *TMC8* transcript levels are much lower in normal and HPV-positive human keratinocytes than in CD4^+^ T-cells suggesting that the levels are too low for anti-viral activity. However, neither the activation of DNA sensing pathways by HPV genomes, supernatants from activated immune cells, nor activation of pathways important for the viral life cycle such as the DNA damage response or keratinocyte differentiation induce high levels of TMC6 or TMC8 in normal keratinocytes. This is consistent with findings that TMC6 and TMC8 do not restrict Mus musculus PV1 expression in keratinocytes. Taken together, we find no evidence for restriction factor activity of EV susceptibility genes for beta-HPV8 or conditions to induce high levels of TMC6 and TMC8 in keratinocytes. Thus, it is plausible that the EV phenotype in humans may be associated with an immune deficiency involving immune cells.

## Highlights

• Epidermodysplasia verruciformis patients are prone to beta-HPV infections.
• EV is caused by mutations in CIB1, TMC6, or TMC8.
• CIB1, TMC6, and TMC8 form a complex hypothesized to inhibit beta-HPV replication.
• Knock-down of CIB1, TMC6, or TMC8 does not impact beta-HPV8 expression.
• TMC8 expression is high in T-cells, but very low in keratinocytes.
• TMC8 expression cannot be activated by pathways important for HPV replication.
• TMC8 expression cannot be activated by molecules secreted from immune cells.
• EV is most likely caused by an immune deficiency.

## 1 Introduction

High risk human papillomaviruses (HPV) from the genus alpha can cause ano-genital and oro-pharyngeal cancers. In contrast, HPV from the genus beta have been implicated in the development of cutaneous squamous cell cancer in *epidermodysplasia verruciformis* (EV) and organ transplant patients [1]. HPV replicate exclusively in keratinocytes, the major cell type in cutaneous and mucosal epidermis and have adapted their replication cycle to the differentiation state of the infected cell: only limited viral genome replication and early gene expression take place in undifferentiated, basal keratinocytes, whereas late gene expression and virion synthesis occur in differentiated, suprabasal keratinocytes.

EV is a rare skin disease inherited autosomal-recessively and characterized by persisting, disseminated benign wart-like skin lesions [1]. Several decades after disease onset, patients frequently develop cutaneous squamous cell carcinoma (cSCC) mainly on sun-exposed sites. The wart-like lesions are mainly positive for beta-HPV, but also alpha- and gamma-HPV can be found [1,2]. CSCC are predominantly positive for beta-HPV from species 1 and 2 [1]. Genetic studies have linked biallelic inactivating germline mutations in one of three human genes to EV: *TMC6*, *TMC8* and *CIB1* [3,4]. *TMC6* encodes Transmembrane channel-like protein 6 or Epidermodysplasia verruciformis protein 1 (EVER1), *TMC8* Transmembrane channel-like protein 8 or Epidermodysplasia verruciformis protein 2 (EVER2), and *CIB1* Calcium and integrin-binding protein 1. It has been suggested that TMC6 and TMC8 control intracellular zinc homeostasis through interaction with the endoplasmic reticulum-resident zinc transporter ZnT1 [[5], [6], [7]]. Furthermore, the modulation of tumour necrosis factor receptor signalling by TMC8 in keratinocytes has been proposed [8,9]. Both human and murine CIB1 has been shown to form a heterotrimeric complex with TMC6 and TMC8 proteins in the endoplasmic reticulum, which results in a stabilization of the three proteins [3,10]. Therefore, genetic depletion of one component should disrupt complex formation. This has resulted in the model that the CIB1-TMC6-TMC8 protein complex is responsible for the EV phenotype [3]. The loss of CIB1 does not affect intracellular free zinc levels or NFkappaB signalling and thus the function of the CIB1-TMC6-TMC8 complex remains unknown [3]. It is currently unclear if the EV phenotype is due to an impaired immune system or if the CIB1-TMC6-TMC8 complex acts to limit beta-HPV replication in keratinocytes. In favour of the first model is that certain primary immunodeficiencies, HIV infection, or immunosuppressive drug regimens used in solid organ transplant recipients can lead to atypical EV and this can be resolved upon restoration of immunity [[11], [12], [13], [14]]. On the other hand, immunological phenotypes of EV patients aside from the increased susceptibility to beta-HPV are very mild which has given rise to the model that the CIB1-TMC6-TMC8 complex acts as a specific restriction factor of beta-HPV replication in keratinocytes [1,3,15]. Recent studies using Mus musculus PV1, which belongs to the pi genus, but shares similarities with beta-HPV, indicated that murine TMC6 and TMC8 do not act as restriction factors for viral gene expression in murine keratinocytes [16,17]. Rather, TMC6^−/−^ and TMC8^−/−^ mice show reduced clearance of MmuPV1 or developed persistent disease without UV immunosuppression in contrast to wild-type mice [16,17].

Nevertheless, it remained possible that the human CIB1-TMC6-TMC8 complex act as restriction factor for beta-HPV in human keratinocytes due to species- and/or PV genus-specific differences. This has not been tested due to a lack of a keratinocyte replication model for beta-HPV. We have previously described that transfected beta-HPV8, 38 and 49 genomes express multiple spliced transcripts in normal human keratinocytes (NHK) over the course of several days [18,19]. The inactivation of the viral E1 replication activator, E2 replication activator/transcription factor, or the E8^E2 replication/transcription repressor modulates viral gene expression suggesting that the viral genomes actively replicate in human keratinocytes [18,19]. Furthermore, differentiation of HPV8-transfected keratinocytes in organotypic cultures increases transcription of the major capsid protein L1, which suggests that the complete replication cycle of beta-HPV takes place in cultured human keratinocytes [18]. HPV8 belongs to species beta 1 and is often found in the lesions of EV patients, whereas no lesions are detected in the normal population [1]. We therefore set out to test if the CIB1-TMC6-TMC8 complex limits HPV8 gene expression in transiently-transfected NHK by siRNA depletion of CIB1-TMC6-TMC8 complex components.

## 2 Materials and methods

### 2.1 Recombinant plasmids

Plasmid pMC-BESPX HPV8 which allows the production of an HPV8 minicircle genome was obtained from M. Ustav and M. Piirsoo, University of Tartu, Estonia. Minicircle genomes were produced in E. coli strain ZYCY10P3S2T as previously described [20]. Plasmids encoding GFP fusion proteins of human TMC6 and TMC8 are based upon pcDNA 3.1 (Thermo Fisher Scientific) and have been previously described [7]. The expression plasmid for human CIB1 has been previously described and was used for the validation of the qPCR primers [3]. Plasmids used as copy number standards for HPV8 transcripts and human PGK1 have been previously described [18].

### 2.2 Cell culture

NHK were isolated from human foreskin and maintained in KSFM media (Thermo Fisher). The procedure was approved by the ethics committee of the medical faculty of the University Tuebingen (6199/2018BO2), and done according to the principles of the Declaration of Helsinki. NHK were seeded in 12 well plates (125.000 cells/well) and media was exchanged after 24 h for 500 μl PBMC-supernatants mixed with 500 μl KSFM or 1 ml KSFM as a negative control. Cells were harvested for RNA-extraction after 8 h or 24 h post medium exchange. The HPV49 E8-and HPV16 wt cell lines were maintained in the presence of Mitomycin C-treated NIH3T3 J2 cells as described previously [18,19,21]. 293T cells were cultured in Dulbecco's Modified Eagle's Medium supplemented with 10 % foetal calf serum and gentamycin. Organotypic cultures of NHK and HPV49 E8-cell lines were grown as previously described and harvested after 16 d [21,22]. Primary CD4^+^ T-cells and peripheral blood mononuclear cells (PBMCs) were isolated from buffy coats (ZKT Tübingen gGmbH, Tübingen, Germany). PBMCs were isolated by density gradient centrifugation using Ficoll-Paque PLUS (GE Healthcare). Briefly, buffy coats were diluted 1:1 with phosphate-buffered saline (PBS) and layered over Ficoll-Paque, followed by centrifugation at 2200 rpm for 45 min at room temperature without brake. The PBMC layer was carefully collected, washed twice with PBS (Gibco), and resuspended in RPMI 1640 GlutaMAX^TM^ Supplement (Gibco) supplemented with 10 % fetal calf serum (Invitrogen) and 100 μg/mL penicillin-streptomycin (Sigma). CD4^+^ T-cells were isolated by negative selection using the RosetteSep CD4^+^ T-cell isolation kit (StemCell Technologies). For expansion and activation isolated CD4^+^ T-cells and PBMCs were cultured in RPMI-1640 supplemented with 2 mM L-glutamine (Merck Millipore), and 10 ng/ml IL-2 (StemCell Technologies). PBMCs were activated with 1 μg/mL phytohemagglutinin (Sigma) for 3 d. Supernatants were harvested by centrifugation at 3200×*g* for 10 min to remove cellular debris, filtered (0.45 μm pore size) to remove cells, and stored at −20 °C until use. Informed blood donors gave their written consent and the use of these cells for research purposes is approved by the ethics committee (IRB) of the University Hospital Tübingen (IRB #860/2023BO2).

### 2.3 Plasmid and siRNA transfection

The following siRNAs were used: siControl (siAllstars negative control; #1027281; Qiagen), siCIB1 (pool: #L-012261-00 or individual siRNAs: # L-012261-05/-06/-07/-08, Horizon Discovery), siTMC6 (#L-016167-00; Horizon Discovery), and siTMC8 (#L-018385-00, Horizon Discovery). For immunoblot analysis of CIB1, NHK (5∗10^5^) were seeded onto 60 mm plates and transfected with 180 pmol siRNA. For the treatment with Mitomycin C, 2.5∗10^5^ NHK were seeded in 6 well plates. NHK were seeded either in 12 well or 6 well plates (10^5^/2∗10^5^) for RNA samples, transfected with 30/60 pmol siRNA for 12/6 well plates with 3/6 μl (12/6 well) RNAiMAX transfection reagent (Thermo Fisher). When transfecting DNA and siRNA into NHK, 60 pmol siRNA was first transfected 24 h after cell seeding in 6 well plates and then 24 h later 1 μg DNA (6 well) was transfected using FuGeneHD. Cells were harvested 3 d post DNA-transfection. For immunoblot analysis 293T cells (1.75∗10^6^) were seeded in 60 mm plates and transfected 24 h later with 3 μg TMC6 or TMC8 expression plasmid using 7.5 μl FuGeneHD (ThermoFisher). Five h after DNA transfection, cells were incubated with 180 pmol siRNA and 18 μl RNAiMAX transfection reagent (Thermo Fisher).

### 2.4 Immunoblot analysis

To detect TMC6- and TMC8-GFP fusion proteins, transfected 293T cells were lysed in RIPA buffer (1 % (v/v) IGEPAL CA-630; 1 % (v/v) sodium deoxycholate; 0.1 % (v/v) SDS, 150 mM sodium chloride, 10 mM sodium phosphate (pH = 7.2); 2 mM EDTA, 50 mM sodium fluoride, protease and phosphatase-inhibitors), heated to 60 °C for 10 min and then sonified. To detect CIB1 or γH2AX protein, NHK were lysed in RIPA buffer and heated to 95 °C for 5 min. Proteins were blotted onto 0.22 μm nitrocellulose membranes (Amersham). Antibodies against alpha-tubulin (mouse, Calbiochem, #CP06, 1:1000) or GAPDH (mouse, Santa Cruz, #sc-32233, 1:2000) served as loading controls. Antibodies to detect CIB1 (rabbit, St Johns Laboratory, #STJ23139, 1:500), GFP (GFP/YFP Living Colors®, mouse, Clontech, #632381, 1:1000) or γH2AX (Phospho-Histone H2A.X (Ser 139), rabbit, Cell signalling, #9718, 1:1000) were incubated over night at 4 °C. Secondary antibodies against mouse or rabbit were diluted 1:15000 in PBS and incubated for 1 h at room temperature (goat anti-rabbit-IRDye680, LI-COR Biotechnology, 926–68071; anti-mouse-IRDye800, LI-COR Biotechnology, 926–32210). Bound antibodies were detected using an Odyssey Fc imager (LI-COR Biotechnology).

### 2.5 Quantitative PCR

RNA was isolated from cells grown in monolayer or organotypic cultures using QIAshredder (Qiagen) and the RNeasy Mini Kit (Qiagen) according to the manufacturer's recommendations. CDNA was synthesized using the QuantiTect RT Kit (Qiagen) and 50 ng cDNA was used per reaction using a LightCycler480 II (Roche) and the LightCycler480 SYBR green Master mix I (Roche). Primer sequences for *PGK1*; *KRT10* andspliced HPV8 (*URR^E2*, *URR^E4*, *E1^E2*, *E1^E4*) have been previously described [18]. The following primer pairs were used for *CDKN1A*/p21.

(F: CTGGGGATGTCCGTCAGAAC; R: GATGTAGAGCGGGCCTTTGA), *IFNB1* (F: ACTGCCTCAAGGACAGGATG; R: AGCCAGGAGGTTCTCAACAA), *CXCL10* (F: AGGAACCTCCAGTCTCAGCA; R: CAAAATTGGCTTGCAGGAAT), *CIB1* (F: CGGCTTAGTGCGTCTGAGAT; R: GGAGAACGGGAGATGACGTG), *TMC6* (F: CACATGAGCACCGTCTTCCT; R: CTTCCTTGTCCTGGTGTCCC), and *TMC8* (F: ACAAGAGCAGCTGTGAGTCC; R: CCAGGGTGCTGAGTAGTTGG). Primers for *CIB1*, *TMC6* and *TMC8* were validated using the corresponding expression plasmids and direct sequencing of the qPCR product. Primers for *CDKN1A* and *CXCL10* were validated by direct sequencing of the qPCR product.

## 3 Results

### 3.1 Cultured human keratinocytes express varying levels of *CIB1*, *TMC6*, and *TMC8* transcripts that can be efficiently depleted by siRNA

**Fig. 1:**
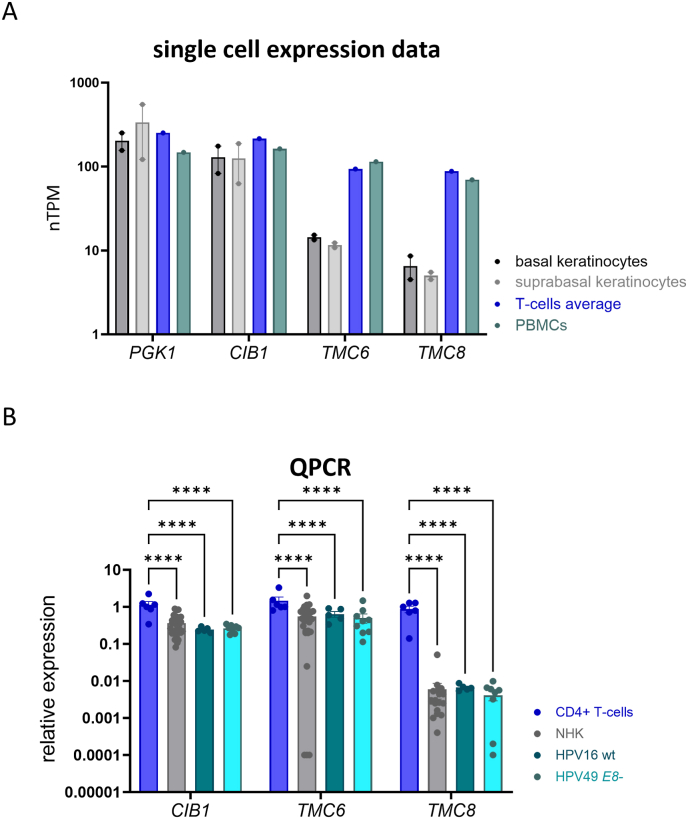
(A) Comparison of normalized transcripts per million (nTPM) values of *CIB1*, *TMC6*, *TMC8,* and *PGK1* obtained from single-cell data provided by the Human Protein Atlas database (https://www.proteinatlas.org/, August 2025). (B) QPCR analysis of *CIB1*, *TMC6*, *TMC8,* and *PGK1* as a reference transcript in activated CD4^+^ T-cells, NHK, HPV16, and HPV49 *E8-*keratinocytes. Data were normalized to one of the CD4^+^ T-cell samples. Statistical significance was determined by ordinary two-way ANOVA using Tukey's multiple comparisons test (∗∗∗∗p < 0.0001). Error bars indicate the SEM.

We first evaluated expression levels of *CIB1*, *TMC6, TMC8,* and *PGK1* (as a house keeping gene) in single-cell RNA sequencing data provided by the Human Protein Atlas database (https://www.proteinatlas.org/). We compared basal and suprabasal keratinocytes from tongue and skin with T-cells and peripheral blood monocytes (PBMC). These data revealed that the levels of *PGK1*, as expected, and CIB1 are similar in basal and suprabasal skin and tongue keratinocytes, T-cells, and PBMCs (Fig. 1A). In contrast, *TMC6* and *TMC8* are expressed at much lower levels in basal and suprabasal keratinocytes than in T-cells and PBMC. We next addressed if HPV modulate *CIB1*, *TMC6*, or *TMC8* levels in keratinocytes. Currently, only immortalized human keratinocyte lines stably maintaining extrachromosomal beta-HPV49 *E8-*genomes (in which the viral E8^E2 repressor is inactivated) are available as HPV8 or HPV38 genomes fail to immortalize NHK [18,19]. We therefore determined *CIB1*, *TMC6* and *TMC8* transcript levels in beta-HPV49 *E8-*keratinocytes, keratinocyte cell lines maintaining extrachromosomal HPV16 genomes, NHK, and activated CD4^+^ T-cells by qPCR analysis (Fig. 1B). This revealed that TMC6 and TMC8 levels are significantly lower in NHK, HPV49 *E8-* -positive, and HPV16-positive keratinocytes than in CD4^+^ T-cells (Fig. 1B). Furthermore, CIB1 expression is also significantly reduced in HPV-positive and -negative keratinocytes. Interestingly, CIB1, TMC6, and TMC8 levels are similar in HPV-positive and HPV-negative keratinocytes suggesting that HPV do not modulate their expression in immortalized keratinocytes (Fig. 1B). Taken together, these data indicate that cultured HPV-positive and -negative keratinocytes express lower *CIB1*, *TMC6*, and, particularly, TMC8 levels than CD4^+^ T-cells.

**Fig. 2:**
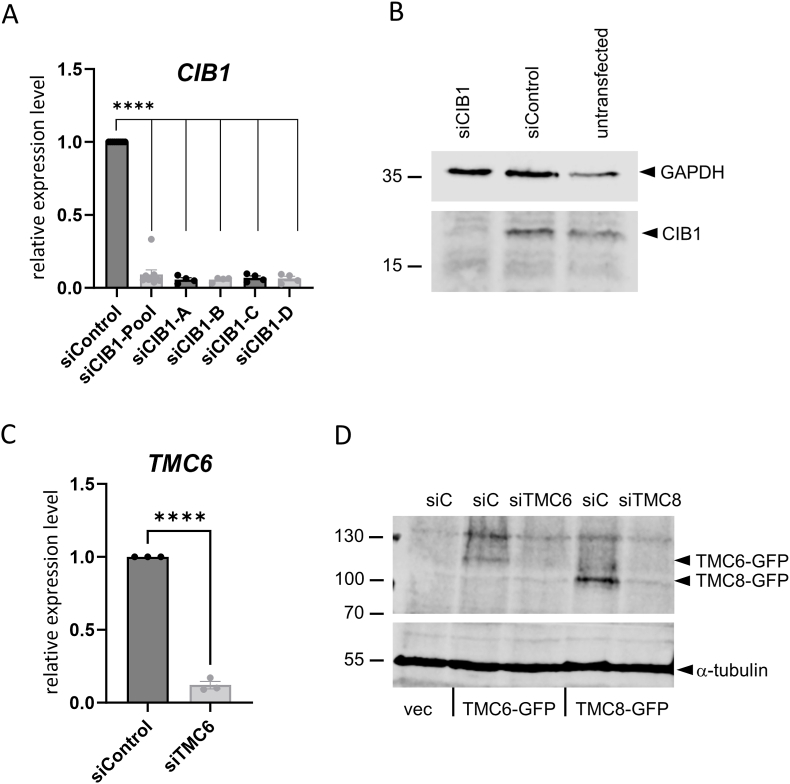
(A) QPCR analysis of *CIB1* transcript levels in NHK 48h post siCIB1-pool transfection. *CIB1* levels were normalized to *PGK1* levels and are shown relative to siControl. Statistical significance was determined using a mixed effects analysis with Dunnett's multiple comparison test (∗∗∗∗p < 0.0001). (B) Immunoblot analysis of CIB1 and GAPDH protein after siRNA transfection in NHK. Molecular size markers are indicated on the left. (C) QPCR analysis of TMC6 transcript levels in NHK after siRNA knockdown for 48h. TMC6 levels were normalized to PGK1 levels and are shown relative to siControl. Statistical significance was determined with a paired *t*-test (∗∗∗∗p < 0.0001). (D) Immunoblot analysis of 293T cells transfected with empty vector (vec.), TMC6-, or TMC8-GFP expression plasmids. Cells were transfected with siControl (siC) or siRNAs against TMC6 or TMC8 5h post plasmid transfection. Whole cell extracts were obtained 48h post siRNA transfection. An anti-GFP/YFP antibody was used to detect GFP fusion proteins and alpha-tubulin was used as a loading control. Molecular size markers are indicated on the left.

Having established that *CIB1*, *TMC6* and *TMC8* are transcribed in keratinocytes, we next tested a siRNA-mediated knock-down of individual complex components for functional studies. We used a siRNA pool against *CIB1* and also the individual siRNAs which revealed that *CIB1* transcripts and protein can be efficiently depleted in NHK (Fig. 2A and B). SiTMC6 depleted efficiently *TMC6* RNA (Fig. 2C). Since reliable anti-TMC6 antibodies are not available, we transfected an expression vector encoding GFP-TMC6 into 293T cells and carried out immunoblot analyses using an anti-GFP antibody. This revealed a specific band of 120 kDa consistent with the predicted molecular weight of GFP-TMC6 that was depleted by siTMC6 (Fig. 2D). The very low TMC8 transcript levels in NHK did not allow determining the effects of siTMC8 by qPCR. Therefore, as described above, an GFP-TMC8 expression vector was transfected into 293T cells and the effects of siTMC8 were analyzed by immunoblot using an anti-GFP antibody. As can be seen siTMC8 depleted a specific band of 110 kDa consistent with the predicted molecular weight of GFP-TMC8 (Fig. 2D). We conclude from these experiments that all complex components can be efficiently depleted by siRNA.

### 3.2 Knock-down of *CIB1*, *TMC6* or *TMC8* does not modulate HPV8 transcript levels in human keratinocytes

**Fig. 3:**
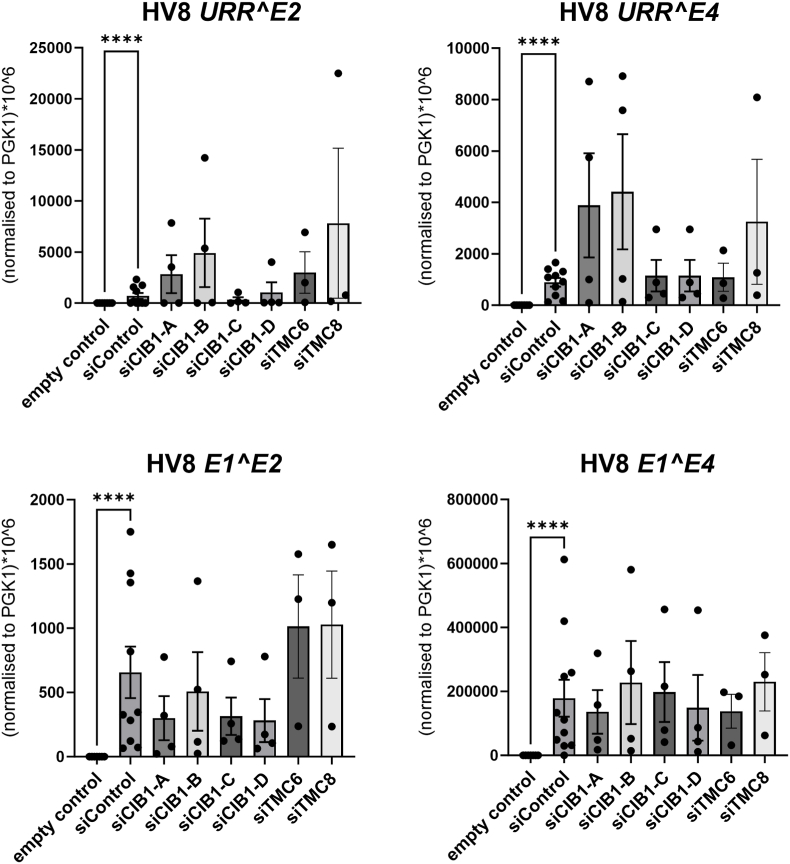
QPCR analysis of spliced HPV8 transcripts (*URR^E2*, *URR^E4*, *E1^E2*, *E1^E4*) using RNA isolated from NHK transfected with HPV8 minicircle plasmids for 3 days and different siRNAs or only empty vector (empty vector) as a negative control. *PGK1* was used for normalization. Statistical significance was determined using a mixed effects analysis with Dunnett's multiple comparisons test using siControl as reference for the other samples. Error bars indicate the SEM.

To determine the effects of CIB1, TMC6 and TMC8 on beta-HPV replication, we used HPV8, a beta-HPV belonging to species 1, often found in EV patients [1]. To enhance transfection efficiency of HPV genomes into NHK, we used supercoiled HPV8 minicircle genomes produced by recombination in bacteria instead of in vitro re-circularized HPV8 genomes [23]. NHK were transfected with HPV8 minicircle genomes and siControl, siCIB1, siTMC6, or siTMC8 and the levels of four spliced viral transcripts (*URR^E2*, *URR^E4*, *E1^E2*, and *E1^E4* [18]) were determined by qPCR 3 days post HPV8 transfection (Fig. 3). NHK transfected with an empty expression vector were used as a negative control for viral gene expression. No viral transcripts could be detected in the absence of the HPV 8 genome (Cp > 40) confirming the specificity of the qPCR assays and the successful expression of viral transcripts from transfected HPV8 genomes. *URR^E2* and *URR^E4* can only derived from the P7535 start site in the upstream regulatory region (URR) of HPV8, whereas *E1^E2* and *E1^E4* transcripts could be derived from different promoters in the URR and the E6/E7 region [24,25]. Initial experiments suggested that the siCIB1 pool of four different siRNAs elevated *URR^E2* and *URR^E4* levels (data not shown). To further explore these findings, we used individual siRNAs from the pool against CIB1 which revealed an efficient knock-down by all individual CIB1 siRNAs (Fig. 2). SiCIB1-A and – B but not -C or- D slightly activated *URR^E2* and *URR^E4* expression, whereas *E1^E2* transcripts were slightly downregulated, and *E1^E4* transcripts were unchanged by all individual CIB1 siRNAs (Fig. 3). However, none of these changes were statistically significant indicating that CIB1 does not strongly influence HPV8 transcription. Similarly, neither siTMC6 nor siTMC8 caused significant changes of *URR^E2, URR^E4*, *E1^E2,* or *E1^E4* transcripts (Fig. 3). In summary, these data do not support the idea that the CIB1-TMC6-TMC8 complex or individual components inhibit HPV8 gene expression.

### 3.3 Neither DNA sensing, DNA damage, keratinocyte differentiation, nor supernatants from activated immune cells induce *TMC8* transcripts

**Fig. 4:**
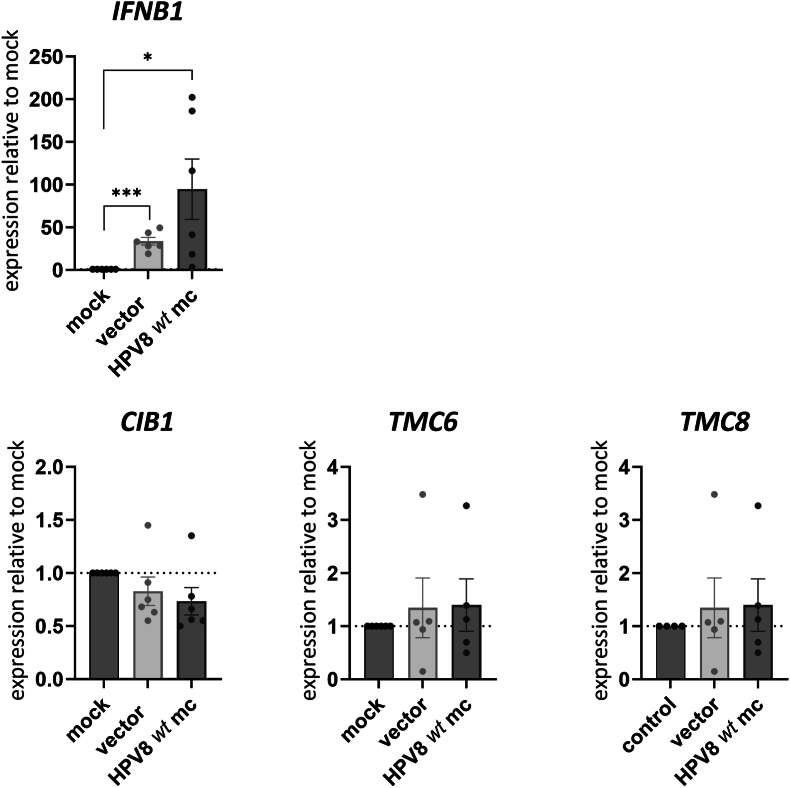
QPCR analysis of *IFNB1*, *CIB1*, *TMC6*, or *TMC8* levels using RNA isolated from NHK non-transfected (mock) or transfected with an empty vector or the HPV8 minicircle genome (HPV8 wt mc) 24 h post transfection. Values were normalized to *PGK1* and calculated relative to the mock control. Statistical significance was determined using paired t-tests (∗p = 0.05; ∗∗∗p = 0.001). Error bars indicate the SEM.

**Fig. 5:**
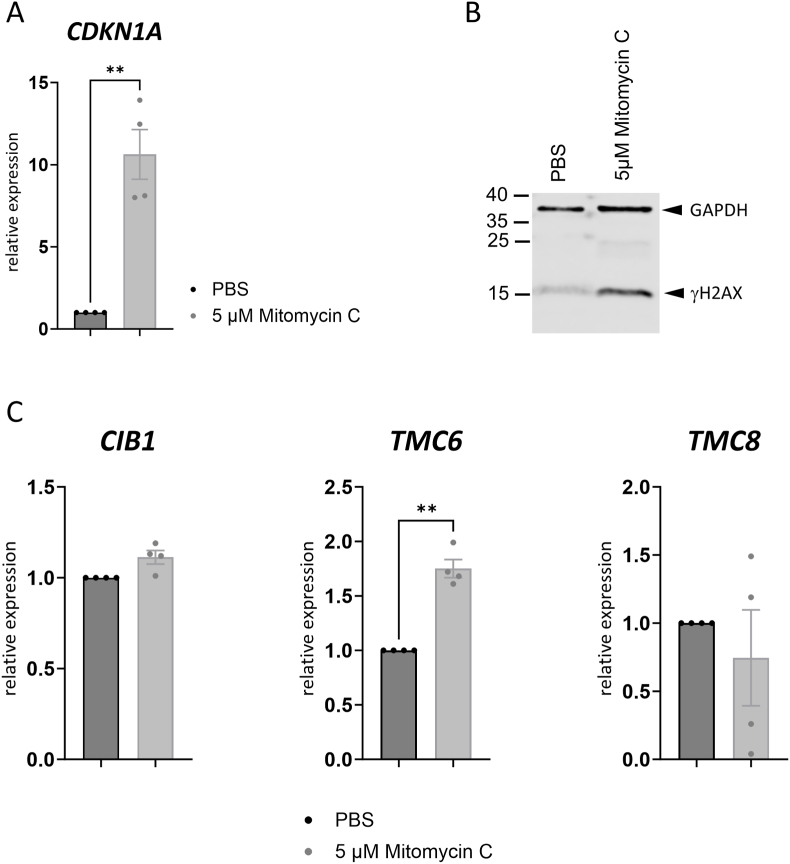
(A; C) QPCR analysis of *CDKN1A*, *CIB1*, *TMC6*, or *TMC8* levels using RNA isolated from NHK incubated with PBS as a negative control or 5 μM Mitomycin C for 24 h. Values were set relative to untransfected control and normalized to *PGK1*. Statistical significance was determined using paired t-tests (∗∗p < 0.01). Error bars indicate the SEM. (B) Immunoblot analysis of γH2AX and GAPDH using protein extracts isolated from NHK incubated for 24 h with PBS or 5 μM Mitomycin C. Molecular size markers in kDa are shown on the left.

It was possible that TMC6 and TMC8 levels are increased in beta-HPV infected keratinocytes by certain stimuli and then inhibitory activity can be detected. We therefore attempted to identify conditions which enhance *TMC6* and *TMC8* transcription in NHK. Transfected DNA has been reported to induce *TMC6* and *TMC8* transcription in the NIKS cell line [26]. Therefore, NHK were either mock-transfected or transfected with an empty expression vector plasmid or HPV8 minicircle genomes and *TMC6* and *TMC8* transcript levels were evaluated 6 h post transfection. Transfected DNA can be sensed by the cGAS/STING pathway which induces interferon-beta (*IFNB1*) transcription [27]. Both transfected DNAs strongly induced *IFNB1* (33- and 94-fold, respectively) suggesting that the cGAS/STING pathway is functional in NHK (Fig. 4). In contrast, neither *TMC6* or *TMC8*, nor *CIB1*were induced by either DNA (Fig. 4). This suggests that DNA sensing does not activate *TMC6*, *TMC8* or *CIB1* expression in NHK, in contrast to the immortalized NIKS cell line [26]. High risk-HPV have been shown to induce a DNA damage response (DDR) which is important for their replication [28]. Furthermore, UV-irradiation, which also induces DNA damage, is an established risk factor for cSCC development [29]. We therefore exposed NHK to 5 μM Mitomycin C, a potent DDR inducer [30]. Consistent with an activation of the DDR, expression of S139-phosphorylated H2A.X (γH2AX) and the p53 downstream target *CDKN1A* (which encodes p21) were induced by Mitomycin C (Fig. 5A and B). However, only *TMC6* transcripts were weakly induced (1.8-fold), but not *CIB1* or *TMC8* transcripts (Fig. 5C). This suggests that *TMC8* is also not a target of the DDR.

**Fig. 6:**
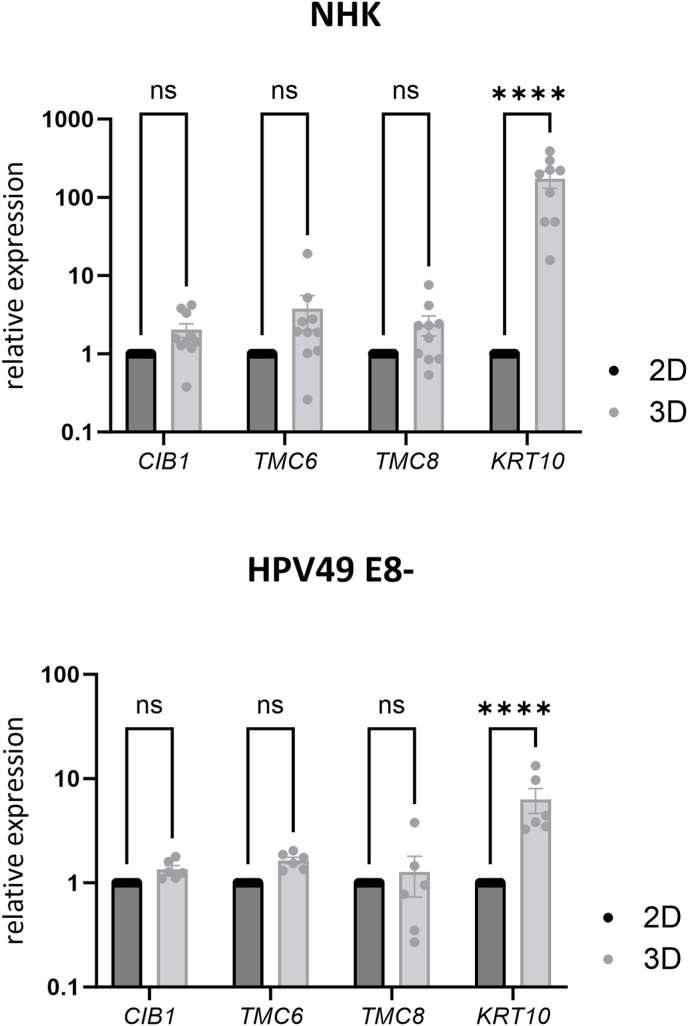
QPCR analysis of *CIB1*, *TMC6*, *TMC8,* and *KRT10* levels using RNA isolated from NHK (upper panel) or HPV49 *E8-*keratinocyte cell lines (lower panel) grown in monolayer cell culture (2D) or organotypic cultures (3D). Values were calculated relative to monolayer cells using *PGK1* for normalization. Statistical significance was determined using a mixed effects analysis with Šídák's multiple comparisons test (∗∗∗∗p = 0.0001). Error bars indicate the SEM.

Keratinocyte differentiation is required to complete the HPV replication cycle. We therefore compared *CIB1*, *TMC6*, and *TMC8* transcript levels in NHK or beta-HPV49 E8-keratinocyte cell lines grown in monolayer culture or in organotypic culture to induce differentiation. Whereas keratin 10 (KRT10), a marker for suprabasal keratinocytes, was significantly induced, neither *CIB1, TMC6* nor *TMC8* expression was activated by keratinocyte differentiation in HPV-negative or beta-HPV-positive keratinocytes (Fig. 6).

**Fig. 7:**
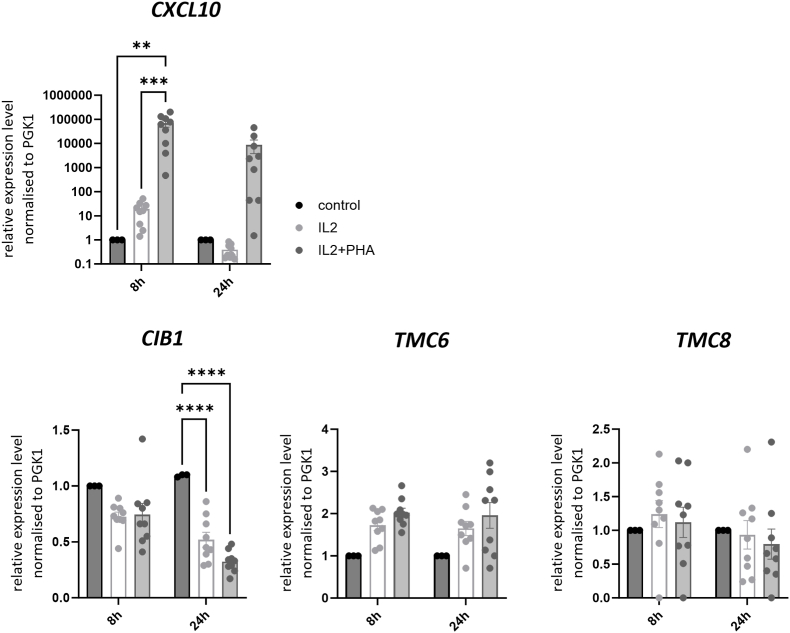
QPCR analysis of *CXCL10*, *CIB1*, *TMC6* and *TMC8*levels using RNA isolated from NHK from three different donors, incubated for 8 h or 24 h with supernatants from PBMCs from three different healthy donors either cultivated with IL2 or IL2 and PHA. Values were calculated relative to control cells (non-treated cells, grown in KSFM-media without supernatant from PBMCs) using *PGK1* for normalization. Statistical significance was determined using a 2 way ANOVA with either Dunnett's multiple comparisons test for *TMC6/8* or Tukey's multiple comparisons test for *CXCL10* (∗∗p < 0.01, ∗∗∗p < 0.001, (∗∗∗∗p = 0.0001). Error bars indicate the SEM.

It was possible that CIB1, TMC6, or TMC8 expression is induced in keratinocytes by ligands secreted by immune cells. To analyze this, PBMC which consist of lymphocytes (T-cells, B-cells and natural killer cells), monocytes and dendritic cells were activated with interleukin 2 (IL2) alone or with a combination of IL2 and phytohemagglutinin (PHA) for 72h. PBMC supernatants were sterile-filtered to avoid contamination of NHK cultures with immune cells, which express high levels of CIB1, TMC6, and *TMC8* (Fig. 1), and then added to NHK from three different donors. RNA was isolated 8 and 24h later and analyzed by qPCR. PHA induces several cytokines including interferon-gamma in PBMC and therefore, the interferon-gamma target gene *CXCL10* was used as a control [[31], [32], [33]]. Supernatants from IL2/PHA-treated PBMC, but not the only IL2-treated PBMC, strongly activated *CXCL10* transcription after 8 and 24h in NHK (Fig. 7). In contrast, neither *TMC6* nor *TMC8* expression was significantly induced by PBMC supernatants, while CIB1 were even significantly decreased after 24h (Fig. 7). Thus, secreted molecules from immune cells isolated from peripheral blood do not induce *TMC6* or *TMC8* expression in keratinocytes. Taken together, neither DNA sensing, DDR induction, keratinocyte differentiation, nor secreted molecules from immune cells induce *TMC6* or *TMC8* expression in cultured keratinocytes.

## 4 Discussion

EV is an autosomal recessive disorder characterized by an extraordinary susceptibility to infections with papillomaviruses, mainly from the genus beta. EV patients carry biallelic loss-of-function mutations in *TMC6* (EVER1), *TMC8* (EVER2) or *CIB1* [3,4]. TMC6, TMC8 and CIB1 form a protein complex in the endoplasmic reticulum which has been hypothesized to act as a restriction factor for beta-HPV infection in keratinocytes [3].

We have tested this model by using cultured human keratinocytes transfected with HPV8 genomes, a beta-HPV commonly found in EV patients, as we have previously described that different beta-HPV genomes are transcriptionally active in keratinocytes for several days [18,19]. Consistent with previous studies, *TMC8* transcripts are very low compared to *CIB1* and *TMC6* in both cultured normal and HPV-positive keratinocytes, whereas *TMC8* is highly expressed in activated CD4^+^ T-cells [5]. SiRNAs targeting *CIB1*, *TMC6*, or *TMC8* greatly decreased the corresponding transcripts and proteins, but did not activate HPV8 transcription challenging the idea that the endogenous CIB1-TMC6-TMC8 complex acts as an anti-beta-HPV restriction factor. We have not tested the contribution of CIB1, TMC6, and TMC8 to productive replication. The clinical phenotype of EV is characterized by multiple, extended, flat, beta-HPV-positive warts [1] which are absent from the immunocompetent population. It is thus possible that EV susceptibility genes are mainly involved in controlling lesion size, which is most likely a consequence of the capability of virus-infected cells to expand in the basal layer, and not of viral entry or virus production.

While TMC6 and particularly TMC8 levels are low in cultured NHK, it remains possible that their expression can be induced by signals to levels that are anti-viral. Currently, very little is known about the transcriptional regulation of TMC6 and TMC8: their expression is high in T-cells and DNA sensing pathways activate their expression in the immortalized NIKS keratinocyte and the myeloma-derived RPMI-8226T cell lines [5,26]. We therefore stimulated DNA recognition pathways in NHK by transfecting an empty expression plasmid or minicircle HPV8 genomes. This induced the positive control *IFNB1*, but not *TMC6* or *TMC8* expression which suggests differences between normal keratinocytes and immortalized cell lines. This finding is also consistent with a lack of induction of *TMC6* and *TMC8* by interferon-beta in NHK [3]. Furthermore, the activation of DNA damage response pathways induced *CDKN1A* transcription and phospho-S139-H2A.X protein expression, but not *CIB1* or *TMC8* expression, and only very weakly *TMC6*. Growth of normal or beta-HPV49-positive keratinocytes in organotypic cultures induced the suprabasal keratinocyte marker *KRT10*, but not CIB1, *TMC6* or *TMC8* expression. Finally, incubation with supernatants derived from stimulated immune cells from peripheral blood, despite being able to induce *CXCL10*, an interferon-gamma target gene, did not induce *TMC6* or *TMC8* transcription in keratinocytes, and furthermore slightly reduced *CIB1* transcription. Taken together, the activation of pathways important for the HPV replication cycle or immune-activated pathways do not induce TMC6 or TMC8 in keratinocytes. While we have not extensively tested potential induction conditions, the restriction of high level TMC8 expression to immune cells may indicate that TMC8 expression is mainly controlled by cell-type specific pathways which could prevent activation in keratinocytes.

Recent publications reported that MmuPV1 gene expression is similar in infected wt and TMC6 or TMC8 knock-out mice and cultured keratinocytes from these mouse strains which resembles our siRNA knock-down experiments [16,17]. This suggests that *TMC8* levels are also not sufficient to repress papillomavirus expression in murine keratinocytes in vivo. Since the lack of the CIB1-TMC6-TMC8 complex causes a subtle cellular immune deficit in mice specific for MmuPV1 [17], it is plausible that the EV phenotype in humans may be associated with an immune deficiency involving immune cells.

## CRediT authorship contribution statement

**Tina M. Rehm:** Writing – original draft, Visualization, Methodology, Investigation, Formal analysis, Data curation, Conceptualization. **Christina Parpoulas:** Investigation, Formal analysis. **Elke Straub:** Methodology, Investigation, Formal analysis. **Thomas Iftner:** Writing – original draft. **Frank Stubenrauch:** Writing – original draft, Visualization, Supervision, Project administration, Investigation, Funding acquisition, Data curation, Conceptualization.

## Funding source

This work was funded by a grant from the 10.13039/501100001659Deutsche Forschungsgemeinschaft (Stu 218/6-1) to F.S.

## Declaration of competing interest

The authors declare that they have no known competing financial interests or personal relationships that could have appeared to influence the work reported in this paper.

## Data Availability

Data will be made available on request.
